# Convolutional mesh autoencoders for the 3-dimensional identification of FGFR-related craniosynostosis

**DOI:** 10.1038/s41598-021-02411-y

**Published:** 2022-02-09

**Authors:** Eimear O’ Sullivan, Lara S. van de Lande, Athanasios Papaioannou, Richard W. F. Breakey, N. Owase Jeelani, Allan Ponniah, Christian Duncan, Silvia Schievano, Roman H. Khonsari, Stefanos Zafeiriou, David. J. Dunaway

**Affiliations:** 1 UCL Great Ormond Street Institute of Child Health, London, UK; 2 Assistance Publique - Hôpitaux de Paris, Faculty of Medicine, University of Paris, Paris, France; 3 Department of Maxillofacial Surgery and Plastic Surgery, Necker - Enfants Malades University Hospital, Paris, France; 4 Department of Computing, Imperial College London, London, UK; 5 Department of Plastic Surgery, Royal. Free Hospital, London, UK; 6 Craniofacial Unit, Alder Hey Childrens Hospital, Liverpool, UK

**Keywords:** Health care, Medical research, Mathematics and computing

## Abstract

Clinical diagnosis of craniofacial anomalies requires expert knowledge. Recent studies have shown that artificial intelligence (AI) based facial analysis can match the diagnostic capabilities of expert clinicians in syndrome identification. In general, these systems use 2D images and analyse texture and colour. They are powerful tools for photographic analysis but are not suitable for use with medical imaging modalities such as ultrasound, MRI or CT, and are unable to take shape information into consideration when making a diagnostic prediction. 3D morphable models (3DMMs), and their recently proposed successors, mesh autoencoders, analyse surface topography rather than texture enabling analysis from photography and all common medical imaging modalities and present an alternative to image-based analysis. We present a craniofacial analysis framework for syndrome identification using Convolutional Mesh Autoencoders (CMAs). The models were trained using 3D photographs of the general population (LSFM and LYHM), computed tomography data (CT) scans from healthy infants and patients with 3 genetically distinct craniofacial syndromes (Muenke, Crouzon, Apert). Machine diagnosis outperformed expert clinical diagnosis with an accuracy of 99.98%, sensitivity of 99.95% and specificity of 100%. The diagnostic precision of this technique supports its potential inclusion in clinical decision support systems. Its reliance on 3D topography characterisation make it suitable for AI assisted diagnosis in medical imaging as well as photographic analysis in the clinical setting.

## Introduction

Early diagnosis of many genetic disorders improves outcome, but rarity and the variety of possible syndromes make clinical diagnosis problematic. Syndromic craniosynostosis (SC) comprises a group of conditions characterised by premature fusion of the skeletal sutures, compromising normal development of the skull and brain^1,2^. Delayed diagnosis is common in phenotypically mild SC risking irreversible functional impairment, including visual failure, neurocognitive defects and airway problems that can be avoided by timely diagnosis and treatment. The inadequacy of current screening paradigms makes syndromic craniosynostosis a prime candidate for computer-assisted diagnosis and referral. Muenke, Apert, and Crouzon syndrome are SC variants caused fibroblast growth factor receptor (FGFR) gene mutations and occur between 1:30,000 to 1:65,000 live births^3^. Phenotypic presentation is variable with some crossover in phenotype between the syndromes.

The application of computer vision and deep learning approaches has already proven effective for the identification and diagnosis of CS from 2-dimensional (2D) images^4–7^. Among the more prominent examples is DeepGestalt, a deep convolutional neural network (DCNN) trained on tens of thousands of images to identify facial phenotypes for genetic disorders^8^. While such systems demonstrate impressive results, they are unable to take advantage of the rich geometric information in the face and cranium that may give critical insight into the phenotypical variations associated with different syndromes. Advances in 3-dimensional (3D) modelling and geometric deep learning have resulted in the introduction of a more shape-based approach craniofacial analysis^9–12^. Previous work has successfully applied statistical shape models in adult populations for diagnosis and surgical simulation in orthognathic patients^13^. A review by Egger et al. 2020 provides an overview on all existing models of which some have been applied beyond faces, such as the surface of the human body and other parts of the body, like the ear and hand^14^. Hammond and their group have performed extensive work on 3D models, however, did not perform diagnostic classifications for FGFR-related syndromes, A study by Hallgrimsson et al. 2020 attempted automated syndrome identification using parametric and machine learning approaches to phenotype the face for nearly 400 syndromes^15^. They could distinguish syndromic cases from unaffected in 80% of their population.

The recent introduction of convolutional mesh autoencoder models (CMAs), a deep neural network approach to 3D model construction, offers further potential for the construction of shape-based models^12,16^. These models learn to extract meaningful shape features from the input data and can consequently be used for classification tasks. Such approaches have not yet been applied for automated diagnosis of craniofacial syndromes using an age matched population and could potentially increase the diagnostic power based on shape information only.

We report on a geometric deep learning approach to the characterisation and identification of SC. The framework leverages convolutional mesh autoencoders and is trained using 3D data from healthy and syndromic individuals, focused on the identification of three distinct types of SC, namely Apert, Crouzon, and Muenke syndrome. Rather than relying on image data, the proposed model leverages the rich geometric information of the 3-dimensional scans. We demonstrate the power of the model for syndrome characterisation and classification, and illustrate its diagnostic sensitivity with an unusual Crouzon case.

## Results

A summary of the demographics for the subjects used in this study is given in Table 1. This includes data from four different databases: CT data from 122 SC patients [Apert, Crouzon, and Muenke, mean age of 5.0 ± 5.1 years, 58% male (n = 70)] and 142 healthy infants (mean age, 1.9 ± 1.2 years, 56% male), Stereophotogrammetric data from 196 healthy subjects from the LSFM dataset^17^, and 139 healthy subjects from the LYHM database^11^. As the volunteers in both datasets were typically above the age of four while many cases in the syndromic dataset were quite young, a dataset of CT scans from healthy children was also used. For full details, see “Methods”.

**Table 1:** Overview of the face and cranium dataset of the included Syndromic Craniosynostosis and normal samples. All syndromic and infant samples were acquired via CT-scan. The LSFM and LYHM databases were obtained using 3dMDphotometric stereo capture device set-ups^10,11^.

Type of SC	Number of subjects	Average age, years	Age range at time of scan	Sex (M:F)
**Face**				
LSFM	196	10.5 ± 4.0	4 years–17 years	98:98 (50%:50%)
Paediatric	142	1.9 ± 1.2	1 day–47 months	79:63 (56%:44%)
Apert	47	6.1 ± 6.2	48 days–20 years	28:19 (60%:40%)
Crouzon	61	5.3 ± 4.4	25 days–17 years	35:25 (58%:42%)
Muenke	14	1.6 ± 2.1	1 day–8 years	7:7 (50%:50%)
Total	460		1 day–20 years	247:213 (54%:46%)
**Cranium**				
LYHM	139	10.9 ± 3.8	4 years–18 years	76:63 (55%:45%)
Paediatric	111	1.8 ± 1.1	1 day–47 months	59:52 (53%:47%)
Apert	39	6.5 ± 6.3	48 days–20 years	22:17 (56%:44%)
Crouzon	53	5.4 ± 4.4	5 months–17 years	30:23 (57%:43%)
Muenke	11	1.7 ± 2.3	1 day–8 years	6:5 (55%:45%)
Total	353		1 day–20 years	193:160 (55%:45%)

### Intrinsic model evaluation

Using the available databases, three distinct classes of model were constructed to assess the role of facial and cranial shape in the diagnosis of SC; a face-only model, a head-only model, and a combined head-and-face model (see “Methods”).

All models reported low reconstruction errors when assessing their reconstruction accuracy and specificity (see “Methods”). For the face, head and combined models, these values were 1.4 ± 1.2 mm, 3.8 ± 3.1 mm, and 2.9 ± 2.5 mm, respectively. Reconstruction error was higher for models that included the head shape, likely due to the greater degree of variation between subjects in this region. Model specificity was evaluated by randomly synthesising 1000 samples and comparing them to their nearest real neighbour^10^. Values of 2.7 mm, 4.3 mm, and 3.9 mm for the face-only, head-only, and combined models respectively indicate that the samples generated are realistic.

### Manifold visualisation

**Figure 1:**
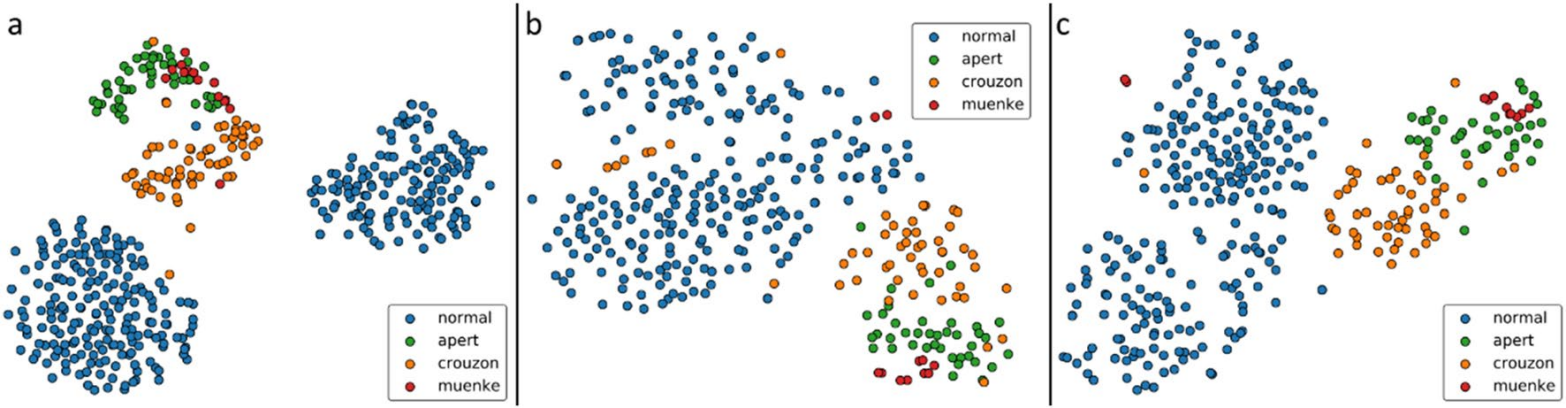
t-SNE embeddings of face-only (**a**), head-only (**b**), and combined head-and-face (**c**) models from left to right respectively. In all cases, distinct clusters emerge for healthy and syndromic samples. The clearest disambiguation between samples is observed for the face-only model.

To assess the diagnostic capacity of the model, t-distributed stochastic neighbour embedding (t-SNE) was applied to the high dimensional latent vectors of the patients and volunteers. (Fig. 1) When samples were labelled by syndromic class, clear clusters emerge for the face-only healthy and syndromic groups (Apert, Crouzon, and Muenke). The two clusters that are observed for the healthy individuals are due to age, with samples from the paediatric and LSFM datasets clustering separately. Within the syndromic cluster, we observe further sub-clusters for the included syndromes. The clusters formed for the head-only embeddings are not as distinct as those observed for the head-only cases, however groups for Apert, Crouzon, Muenke, and healthy individuals do emerge. When considering the model constructed using the combined head-and-face template, we again see clear clusters forming for the different subgroups in the dataset.

In all cases, even though the syndromic samples tend to group more tightly together, it is noted that the syndromes themselves seem relatively disentangled. The proximity of the Crouzon cluster to the normal cases in each of the embeddings indicates that this phenotype has a milder manifestation than either Apert or Muenke syndrome. For both the head-only and combined head-and-face embeddings, we note that a number of Muenke and Crouzon samples cluster closer to the group of healthy cases.

### Syndrome classification

**Figure 2:**
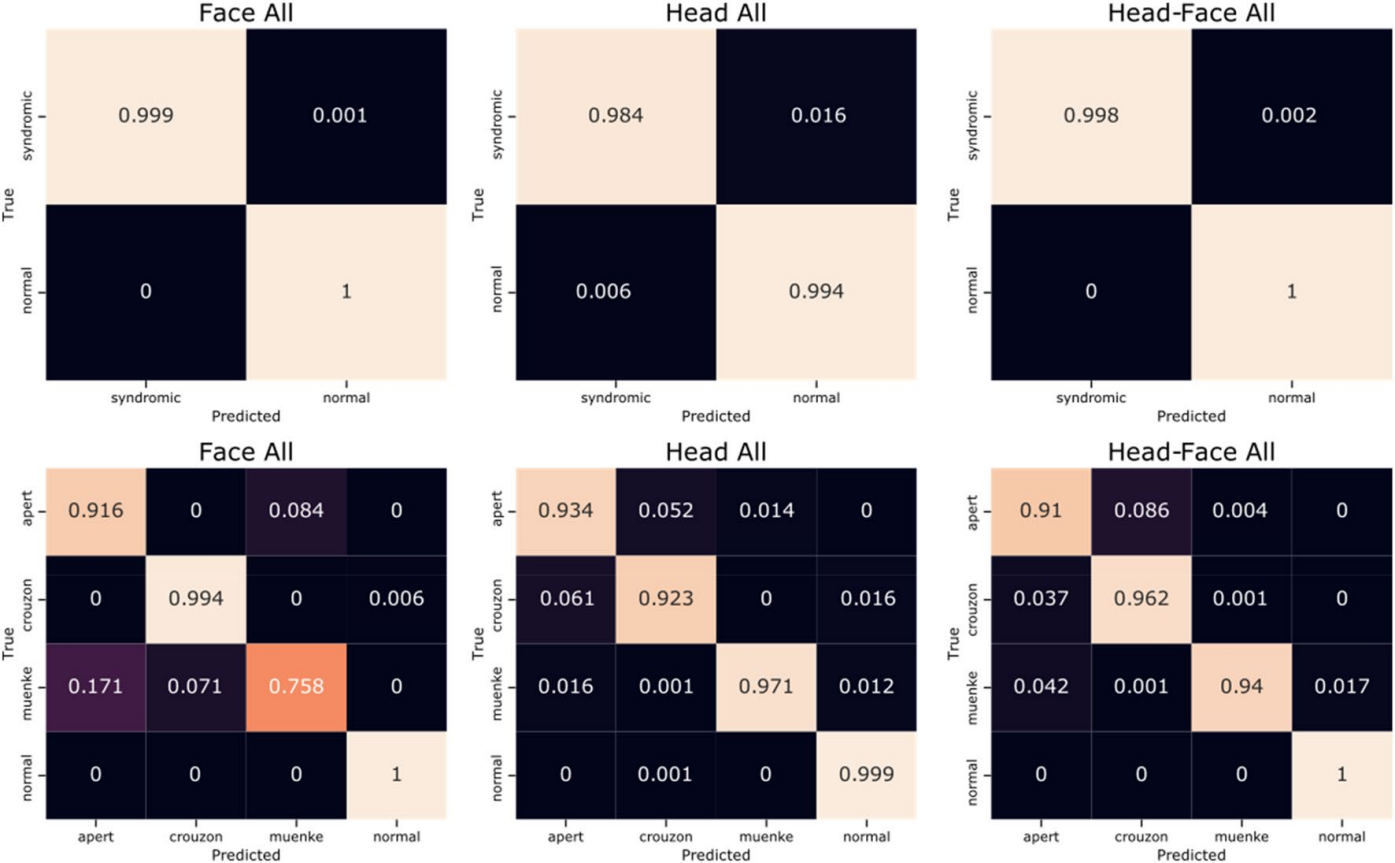
Confusion matrices for the face-only, head-only, and combined head-and-face models in order from left to right. Top row: binary classification. Bottom row: multi-class classification.

Classification was performed with all syndromic and non-syndromic scans. A split of 80–20% for training and testing data was assessed over 1000 iterations. The mean sensitivity, specificity, and accuracy over all iterations for each of the assessed regions in the binary classification experiment, and the confusion matrices for both binary and multi-class classification, are shown in Table 2 and Fig. 2, respectively.

**Table 2:** Classification results for the binary classification experiments.

Model	Sensitivity (%)	Specificity (%)	Accuracy (%)
Face only	99.95	100.00	99.98
Head only	98.36	99.41	99.09
Head and face	99.82	100.00	99.95

Binary classification to identify whether an individual belonged to either the syndromic or non-syndromic groups showed accuracies of greater than 99% in all cases. The high sensitivity of the models indicates that very few syndromic cases were misidentified as normal volunteers (one, sixteen, and two cases per thousand for the face-only, head-only, and combined models respectively). The inverse is also true; as indicated by the high model specificity values, few healthy volunteers were seldom, if ever, misidentified as having a craniofacial syndrome.

The multi-class classification model endeavoured to predict whether a patient belonged to either the non-syndromic, Apert, Crouzon, or Muenke categories. Accuracies of 98.3%, 97.9%, and 98.2% were observed for the face-only, head-only, and combined regions, respectively. When considering the face-only, Muenke patients were the most likely to be mis-diagnosed by our model. As there are the fewest instances of this syndrome in the database, such results are to be expected, and increasing the quantity of Muenke samples would likely lead to increased accuracy for these patients. When the head shape was considered for classification, Crouzon and Apert patients were most likely to be misidentified as each other. As with the binary classification, the poorest performance was seen for the head-only model. These results would indicate that the facial region contains valuable shape information for the correct diagnosis of SC.

**Figure 3:**
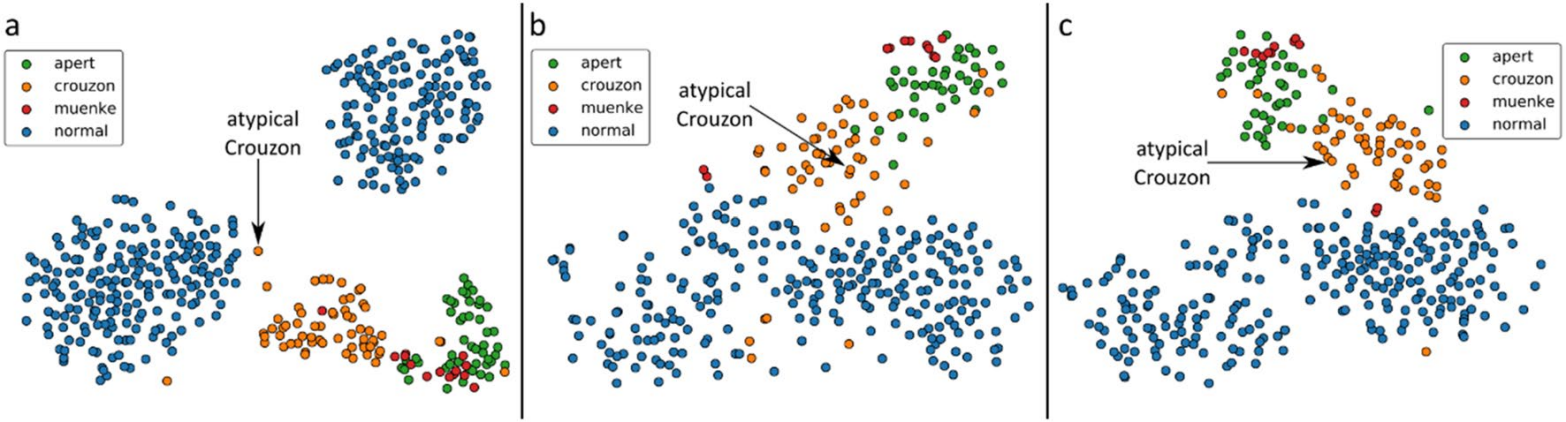
t-SNE embeddings for all samples, including the atypical Crouzon case, for the face-only (**a**), head-only (**b**), and combined head-and-face (**c**) models.

**Figure 4:**
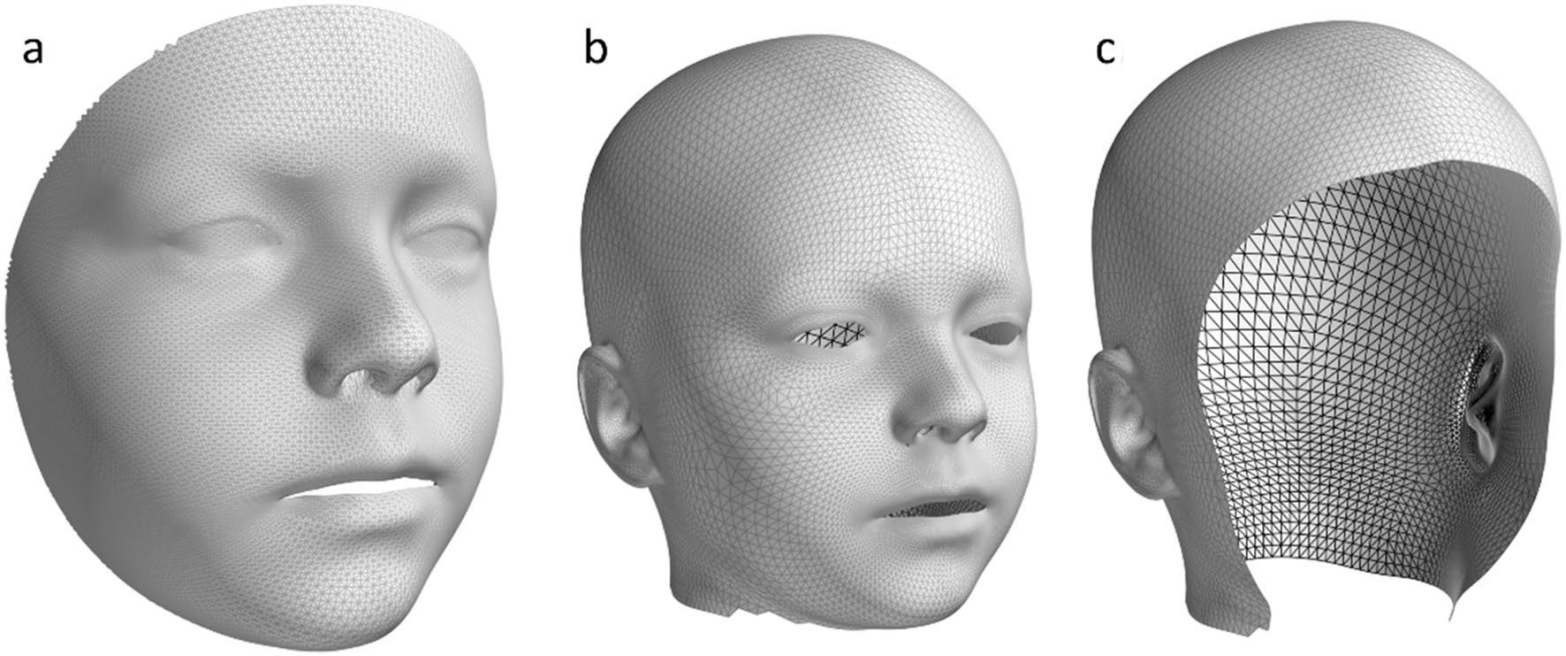
Mesh templates. (**a**) Face only template, (**b**) Head and Face template, (**c**) Head only template.

Our method has proven as least as sensitive as expert diagnosis in a multidisciplinary clinic and in one case more sensitive. A 2-year-old sibling of a patient with Crouzon syndrome, judged clinically to be unaffected, proved to have Crouzon syndrome on genetic testing. Subsequent three images analysed by our technique demonstrated the ability of the model to diagnose clinically undetectable variations from the norm. (Fig. 3) A CT-scan of the child was processed following the pipeline described in the “Methods” section and shape vectors for the child were acquired. All shape vectors were then embedded into a 2D space using t-SNE. When considering the face-only, the atypical instance was embedded at the intersection of the healthy individuals and the Crouzon cases, as shown in Fig. 4a. When the head only was considered, the atypical instance clustered among the Crouzon cases. The same was observed when head and face were jointly considered. Classification experiments were also performed to determine how the atypical case was perceived by an SVM classifier. In the case of multi-class classification, the child was identified as a Crouzon case by all three models. In the binary classification experiments, the child was correctly classified by both the face-only and combined head-and-face models but was incorrectly labelled as a non-syndromic individual by the head-only binary classification model. This supports our earlier observation that facial shape is more characteristic for the included craniofacial syndromes than head shape.

## Discussion

AI assisted diagnosis is set to play and increasing role in healthcare particularly in relation to rare or difficult to diagnose conditions. In this work, we leverage state-of-the-art geometric deep learning approaches to present a framework for the detection and classification of a subset of syndromic craniosynostoses. Requiring only a 3D input allows potentially deceptive texture information to be omitted, allowing the architecture to focus on the extraction of characteristic shape-based features to return an accurate diagnosis.

Our autoencoder model demonstrates high sensitivity and specificity. The model can be applied for both binary classification (syndromic vs healthy) and multi-class classification (Apert, Crouzon, Muenke, and healthy). The high degree of separation of the different classes in the shape space of the model, as demonstrated by the t-SNE embeddings, intuitively supports these results. The high sensitivity and specificity of the model make it suitable as a diagnostic aid in primary and secondary care settings ensuring reliable diagnosis with few false positive results. A possible confounding factor for these results is the mix of 3D image acquisition modalities, though some studies have suggested that there is strong agreement between different shape acquisition systems^18^. All paediatric and syndromic scans were captured using CT imaging, however the over-4 normative data was acquired using surface scanning modalities. That said, a strong separation between the different classes is observed for the under-4 data subset, and multi-class and binary classification accuracies for this subset are comparable to that of the full dataset (Supplementary Materials). This indicates that it is not just the acquisition modality that is driving the classification, but that the model is using shape-based features during prediction. Furthermore, the scans were registered to a common template and analysis was conducted on the registered samples and not on the raw data.

A limitation of this study is the small number of phenotypes that could be included in the analysis. 3D data are less abundant than 2D images and require more processing to be readied for computational analysis than their 2D counterparts. For these reasons, current 3D dataset sizes are constrained.

Utilising 3D topography rather than the surface texture analysis central to most other facial analysis techniques lends itself to integration with many forms of diagnostic imaging technologies, such as MRI, ultrasound scans, and CT. Our technique facilitates auto- segmentation of surfaces as well as syndrome identification and could therefore be used as a machine learning diagnostic tool to aid radiological diagnosis. Integration with ultrasound imaging is of particular interest, as this presents an opportunity for the foetal detection of genetic disorders^19^. The rising availability of 3D scanning applications and cameras on mobile devices presents further possibilities to introduce such a framework in primary and secondary care. In a field where timely diagnosis is necessary for appropriate management, the use of such technologies to detect SC and other conditions will streamline assessment at an earlier stage could be pivotal to improving long-term health outcomes.

While several machine learning approaches for the identification of craniofacial syndromes have been presented 11, to the best of our knowledge, this is the first time the problem of syndrome classification has been approached from a shape-based perspective.

## Methods

### Institutional review board statement

Patient data for this study were retrospectively retrieved from electronic medical records after receiving approval from the Institutional Review Board: Great Ormond Street Hospital (R&D no. 14DS25). Informed consent was obtained from all participants, or if participants are under 18, from a parent and/or legal guardian. All methods were carried out in accordance with relevant guidelines and regulations.

### Data sources

A full summary of the demographics for the databases used is given in Table 1.

All SC patients diagnosed with Apert, Crouzon, or Muenke syndrome at the *Craniofacial Unit of Great Ormond Street Hospital for Children, London, UK* were reviewed retrospectively for preoperative 3D imaging. Computed Tomography (CT) head scans were selected as the most suitable image modality for the assessment of the craniofacial anatomy. CT-scans with insufficient quality for 3D reconstruction due to low number of slices or (movement) artefacts were excluded. Baseline characteristics were collected from corresponding medical charts. We refer to this database as “the syndromic dataset”.

At *Necker Children’s Hospital, (Necker-Enfants Malades Hospital), Paris, France*, CT-scans of patients between the age of 0–4 years without a history of craniofacial anomalies were assessed. Patients indicated for a CT-scan between 2011 and 2018 due to headaches, trauma or epilepsy were reviewed for inclusion. The scans were evaluated by two independent reviewers: (1) a paediatric radiologist and (2) a clinical research fellow in craniofacial surgery, to exclude any scans with abnormalities such as fractures, brain tumours, brain damage or craniofacial anomalies. Henceforth, this database of normal craniofacial images will be referred to as ‘the paediatric dataset’. For the paediatric and syndromic datasets, several samples used for the face models were omitted when constructing the head models. Scans with incomplete cranium information or those with previous calvarial surgery, such as posterior vault remodelling, were excluded.

The original data consisting of 10,000 faces of the Large Scale Face Model (LSFM) was used for age-matched diagnostics for cases between the ages of 4 and 17. Of the subjects who met the desired age criteria, 196 samples were randomly selected to provide this age matched reference (7 male, 7 female for each age).

The Liverpool-York head model (LYHM) was constructed using the craniofacial scans from approximately 1200 individuals aged between 2 and 90 from the Headspace database^11^. The scans from those aged between of 4 and 17 were used to provide an age-matched reference for full head scans from the Syndromic Craniosynostosis dataset. The database included 139 scans that met the required age criteria. The mean age was 10.9 ± 4.4 years, and 55% were male.

### Image pre-processing

#### CT-scan pre-processing

The DICOM-files of the collected datasets, i.e. the syndromic and paediatric datasets were converted to 3D soft tissue meshes by applying standardised skin setting using Horos, an open-source medical viewer, and exported at stereolithography (STL) files. The STL files were imported in MeshMixer, an open-source software, to undergo a cleaning process where redundant objects, such as draping and gel pads, back of the CT-scanner, pacifiers, and lines and tubes, were removed. The meshes were saved as an Object (OBJ) files. A sparse set of 68 facial landmarks were manually added to the meshes. To encourage good correspondence around the ears, an additional 55 landmarks per ear were also added to the meshes, following the landmark template outlined in Ref.^20^. Using the facial landmarks, the raw meshes were subjected to Procrustes analysis, allowing them to be rigidly aligned with the template meshes prior to obtaining dense correspondence.

The construction of a 3D mesh autoencoder model requires that all meshes are re-parametrised to have a consistent topological structure, where each mesh has the same number of vertices connected in consistent triangulation, with similar vertices having the same semantic meaning. Meshes meeting this criterion are said to be in dense correspondence. In this study approach, a non-rigid iterative closest point registration (non-rigid ICP) was used to achieve dense correspondence^21^. This approach allows for the use of landmark points to guide the correspondence process, and the use of data weights to restrict how certain vertices can move during the registration. As this method requires an initial template mesh with the desired final topology, the cropped facial template from Ref.^17^ was used for construction of the facial models, while the template from Ref.^22^ was used for the craniofacial models. (Fig. 4) The head-only template was created by removing the facial region of the combined head-and-face template.

#### Dense correspondence for the Syndromic Craniosynostosis dataset

The faces and heads of those in the infant and syndromic craniosynostosis datasets differ greatly in shape both from each other, and from the samples used for the construction of previous (cranio)facial 3DMMs. For this reason, the use of landmark guided NICP was not always sufficient to achieve good quality correspondences. As such, Gaussian Processes were applied to the face template mesh to increase deformation flexibility and improve the quality of the correspondences obtained for the syndromic cases.

### 3D mesh autoencoder construction

Once dense correspondence had been achieved for all meshes, the 3D models were created using mesh autoencoders. Three classes of model were created: face-only, head-only, and combined head and face models (Fig. 4). For each of these classes, a model was trained using all samples up to and including the age of three, all samples above the age of three, and a final model constructed from all available data.

The autoencoder architecture applied here is similar to that described in Ref.^12^. Four convolutional and down-sampling/up-sampling layers were used for the encoder and decoder for construction of the head-only and combined head-and-face models. Five such layers were used for the face-only model as the template mesh for this model is far denser (28,431 vertices for the face-only model, compared to 7,505 vertices in the head-only model, and 17,039 vertices in the combined model). Encoder convolutional filter sizes of [16, 16, 32, 32, 32] were used for the face-only model, while encoder filter sizes of [16, 16, 32, 32] were used for the head-only and combined models. In both cases, decoder filter sizes were the mirror of the encoder. Each convolutional layer in the encoder was followed by a mesh downsampling layer by a factor of 4—in the decoder, this was replaced with a layer to upsample the mesh by a factor of 4. An additional convolutional layer with an output dimension of 3 is added to the decoder to allow for the reconstruction of the 3D shape coordinates. An ELU activation function was applied after each convolutional layer. Spiral convolutional with a fixed length of nine were applied in all layers^23^, and a latent vector size of 128 was used. A latent vector size of 128 was chosen as it outperformed sizes 16, 32 and 64 in our prior experiments. Model weights were initialised using Xavier initialistion. Adam optimisation with an initial learning rate of 1 × 10^–3^, and a learning rate decay of 0.99 was used. Mesh vertices were used as the autoencoder input and an L1 reconstruction loss was applied to the output. All models were trained for 300 epochs using a batch size of 16.

Models were constructed for the face-only, head-only, and combined head and face regions to gain an insight into how characteristic these regions are of the various craniofacial syndromes. All paediatric healthy cases and syndromic patients with incomplete or poor-quality head CT-scans were omitted from the head-only and combined head and face models. Consequently, these models were constructed using fewer samples. Due to the wide age range of the individuals in the dataset (1 day to 20 years), two additional models were created for each of the regions of interest. The first included all patients and volunteers who were up to and including three years of age. The second consisted of those above the age of three. Nine models were created in total. All results pertaining the age-based models can be found in the supplementary materials.

### Error quantification

To assess reconstruction accuracy, the Euclidean distance, *d*, between each sample in the real dataset and the corresponding model reconstruction was calculated on a per-vertex basis. For two meshes, *A* and *B*, with *n* vertices, the mean error over all vertices is defined as:

$$d=\frac{{\sum }_{i=1}^{n}\sqrt{{\left({x}_{i, A}- {x}_{i, B}\right)}^{2}+{\left({y}_{i, A}- {y}_{i, B}\right)}^{2}+{\left({z}_{i, A}- {z}_{i, B}\right)}^{2} }}{\mathrm{n}},$$

where *x*, *y*, and *z* correspond to the cartesian coordinates of the mesh vertices. The mean error over all samples was reported.

### Model specificity

Specificity is a metric that is commonly used evaluate the validity of novel instances create by generative models. To assess this, 1000 samples were randomly synthesised for each of the models and the Euclidean distance to all ground truth samples was calculated. The specificity error was reported as the mean Euclidean distance over all vertices between a synthesised face and the closest ground truth neighbour.

### Manifold visualisation

t-Distributed Stochastic Neighbour Embedding (t-SNE) is a dimensionality reduction technique that allows the high dimensional shape vectors to be embedded in a lower dimensional space and can reveal hidden structures of the data^24^. To assess the diagnostic capacity of the models, t-SNE was applied to the high dimension latent vector encodings for the patients and healthy volunteers, allowing the global manifold of these vectors to be embedded in a 2-dimensional space for visualisation. Samples were then labelled according to their syndromic class (Normal, Apert, Crouzon, or Muenke) with the aim of uncovering distinct groupings, or clusters. All t-SNE embeddings were created using a perplexity of 30 and run for 1000 iterations.

### Classification

Autoencoders are often utilised for their ability to compress data into a much more compact format. This manifests as the latent vectors of the model. These latent vectors provide a natural means by which we can attempt to classify the data and determine its applicability as a diagnostic tool.

Classification was performed using a Support Vector Machine (SVM) with linear kernel and balanced class weighting. A stratified data split with an 80%:20% train:test proportion was used. The scikit-learn SVM implementation with default gamma and regularization parameters (C = 1.0) was employed. The mean accuracy, specificity, and sensitivity were calculated following a Monte-Carlo cross-validation system where the training and test sets were randomly selected 10,000 times.

## Supplementary Information

Supplementary Information.
