# Supplementary material for: Convolutional mesh autoencoders for the 3-dimensional identification of FGFR-related craniosynostosis
Source: Sci Rep. 2022 Feb 9;12:2230. doi: 10.1038/s41598-021-02411-y (PMC8828904; doi:10.1038/s41598-021-02411-y)
Supplement: Supplementary file 1 — Supplementary Information. [file 41598_2021_2411_MOESM1_ESM.docx]

Convolutional Mesh Autoencoders for the 3-Dimensional Identification of FGFR-Related Craniosynostosis

**Eimear O’ Sullivan, MRes^1,3^**^†^**, Lara S. van de Lande, MD^1,2^**^†^**, Athanasios Papaioannou, MSc PhD^1,3^, Richard W.F. Breakey, MBBS, N. Owase Jeelani, MBa, MPhil (Medical law), FRCS (Neuro.Surg)^1^, Allan Ponniah FRCS (plast)^4^ , Christian Duncan MPhil, FRCSI(Plast)^5^**, **Silvia Schievano, MRes PhD^1^, Roman H. Khonsari MD PhD^2^, Stefanos Zafeiriou, PhD^3^**^§^ **David. J. Dunaway CBE, FDSRCS, FRCS (plast)^1^**^§^

1. UCL Great Ormond Street Institute of Child Health, London, UK & Craniofacial Unit, Great Ormond Street Hospital for Children, London, UK.
2. Oral and Maxillofacial Surgery Department, Hospital Necker, Enfants Malades, Paris, France.
3. Department of Computing, Imperial College London, London, UK.
4. Department. Of Plastic Surgery, Royal. Free Hospital, London, UK
5. Craniofacial Unit, Alder Hey Childrens Hospital, Liverpool, UK

^†^ These authors contributed equally to this work.

^§^ These authors contributed equally to this work

Corresponding author: Eimear O’ Sullivan

* Email: e.o-sullivan16@imperial.ac.uk

# **Supplementary Figures**


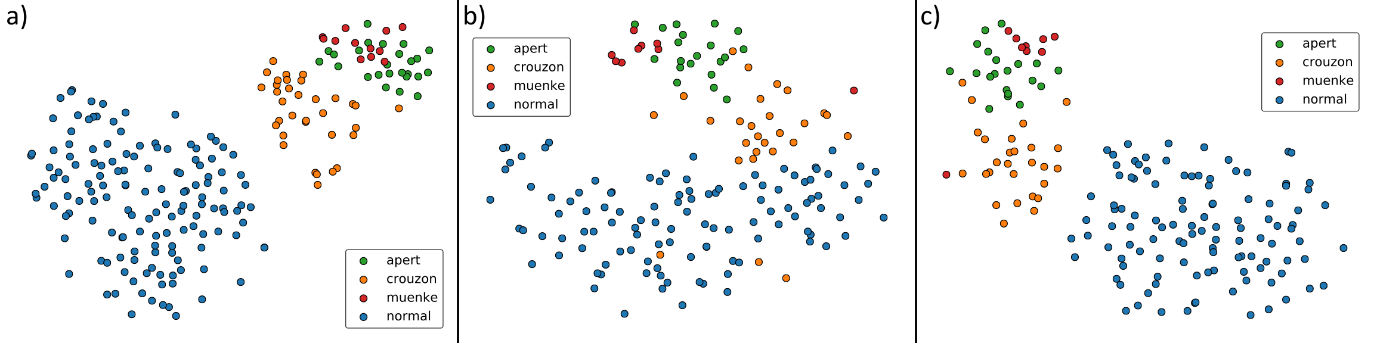


Figure 1: t-SNE embeddings of face-only (a), head-only (b), and combined head-and-face (c) Under-4 models from left to right respectively. In all cases, distinct clusters emerge for healthy and syndromic samples.


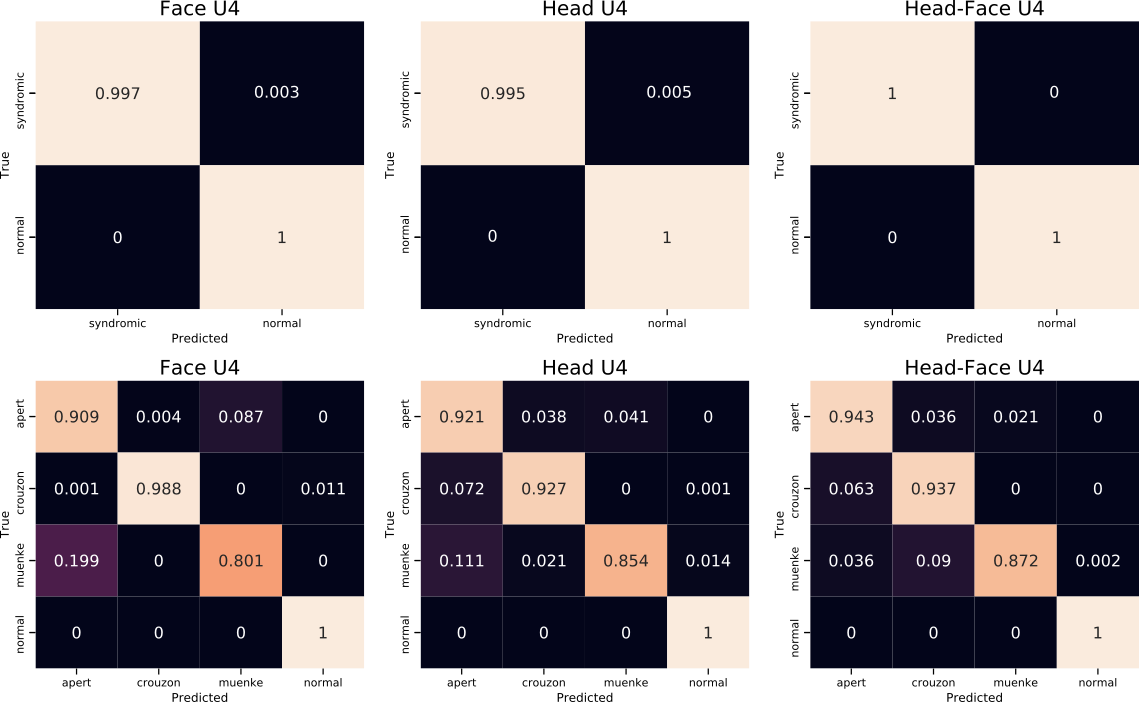


Figure 2: Confusion matrices for the face-only, head-only, and combined head-and-face Under-4 models in order from left to right. Top row: Binary classification. Bottom row: Multi-class classification.


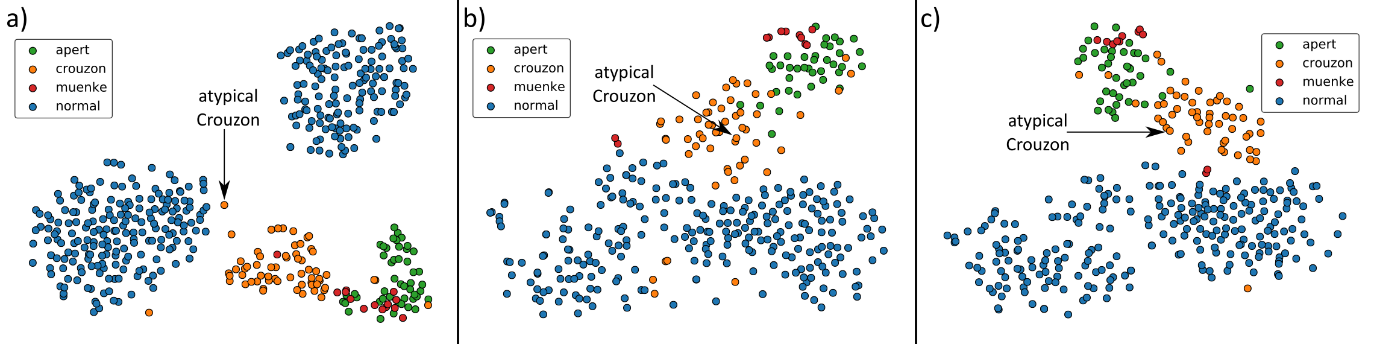


Figure 3: t-SNE embeddings for all samples, including the atypical Crouzon case, for the face-only (a), head-only (b), and combined head-and-face (c) Under-4 models.


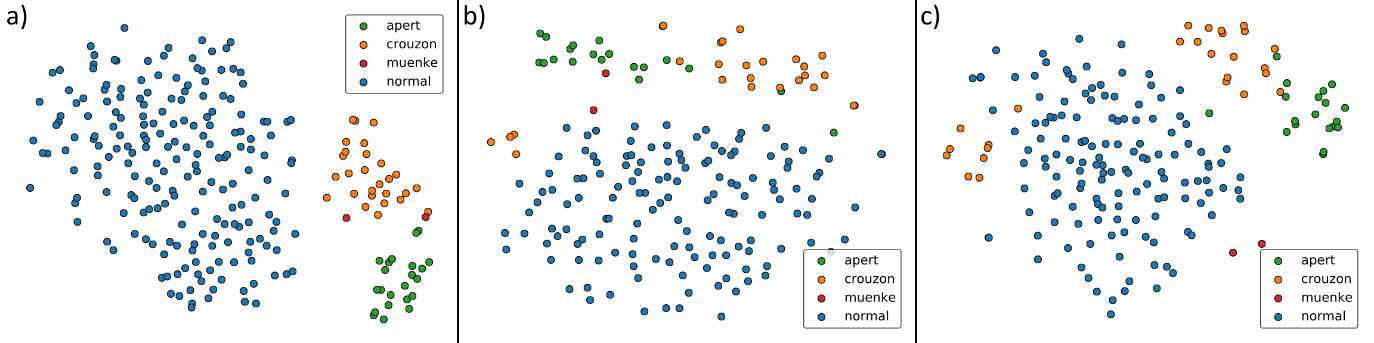


Figure 4: t-SNE embeddings of face-only (a), head-only (b), and combined head-and-face (c) Over-4 models from left to right respectively. In all cases, distinct clusters emerge for healthy and syndromic samples.


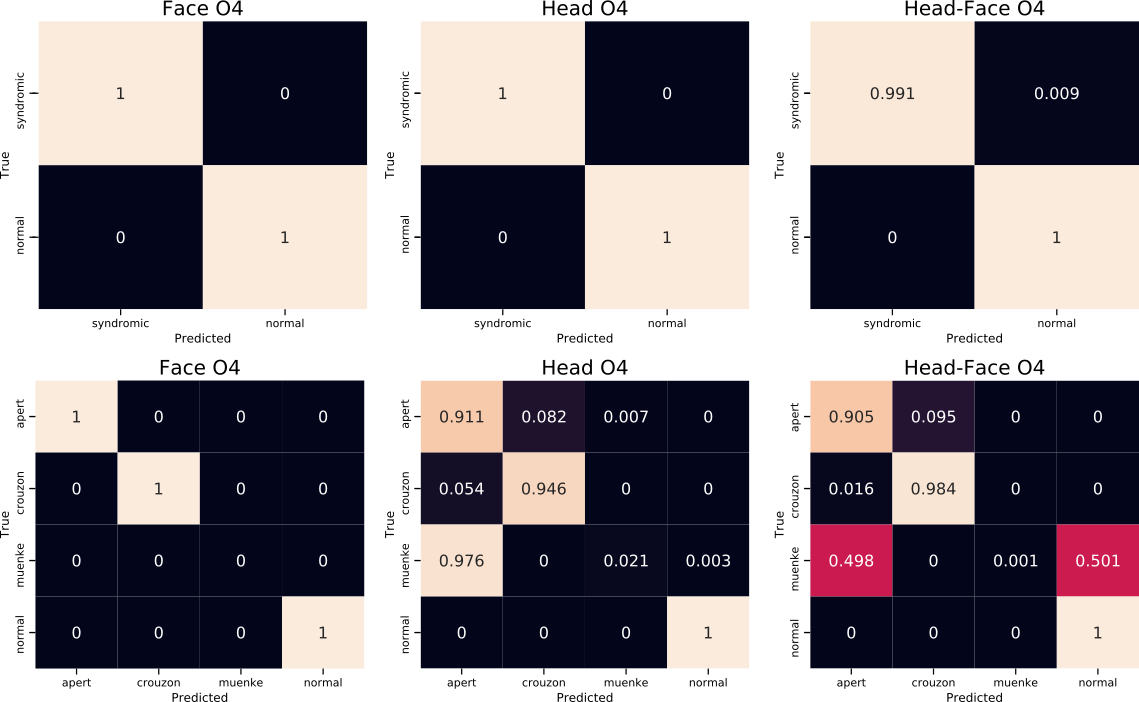


Figure 5: Confusion matrices for the face-only, head-only, and combined head-and-face models in order from left to right. Top row: Binary classification. Bottom row: Multi-class classification.

# **Supplementary Tables**

| Model | Sensitivity (%) | Specificity (%) | Accuracy (%) |
| --- | --- | --- | --- |
| Face Only | 99.71 | 100.00 | 99.90 |
| Head Only | 99.53 | 99.99 | 99.84 |
| Head and Face | 99.95 | 100.00 | 99.98 |

*Table 1. Classification results for the binary classification experiments for all Under-4 models.*

| Model | Sensitivity (%) | Specificity (%) | Accuracy (%) |
| --- | --- | --- | --- |
| Face Only | 100.00 | 100.00 | 100.00 |
| Head Only | 100.00 | 100.00 | 100.00 |
| Head and Face | 99.15 | 100.00 | 99.77 |

*Table 2. Classification results for the binary classification experiments.*

# **Supplementary Materials**

## **Bespoke Models**

Given the large age range of the dataset participants, separate models were constructed for those up to and including the age of 3, and those above the age of three. These are henceforth referred to as the Under-4 and Over-4 models, respectively, while the model constructed using all samples is referred to as the complete model. As with the complete model, a face-only, a head-only, and a combined head-and-face model were constructed for the different age groups (see Methods). In all cases, the methodology for intrinsic model evaluation, manifold visualisation, and syndrome classification is identical to that outlined in the Methods Section.

## **Under-4 Model Intrinsic Evaluation**

Low reconstruction errors were recorded for all models (see Methods). For the face, head and combined models, these values were 1.9 ± 1.6mm, 4.3 ± 3.4mm, and 3.0 ± 2.6mm, respectively. Model specificity values of 2.9mm, 5.2mm, and 4.2mm for the face-only, head-only, and combined models, respectively. These values are higher than those recorded for the complete model, however this is to be expected, given the reduced number of samples used for model construction.

## **Under-4 Manifold Visualisation**

t-SNE manifold visualisation of the U4 populations shows distinct groups forming for the assessed syndromes and the normal population for each of the models. In all cases the Crouzon cases cluster closest to the normal population, indicating that the physical manifestation of Crouzon syndrome is milder than that of either Apert or Muenke syndrome (Figure 1).

## **Under-4 Syndrome Classification**

Binary syndrome classification for the Under-4 models yields accuracies of greater than 99% in all cases (Table 1). As with the complete models, the high model sensitivity indicates that few syndromic cases are misidentified as normal volunteers. The high specificity values demonstrate the normal volunteers are also highly unlikely to be misidentified as a syndromic patient.

Multi-class classification accuracies of 97.8%, 97.1%, and 97.7% were observed for the Under-4 face-only, head-only, and combined head and face models, respectively. These values are marginally lower than those of the combined model, a phenomenon that can likely be attributed to the reduced number of samples used. Confusion matrices for the binary and multi-class classification for all Under-4 models are shown in Figure 2.

When the mild, or atypical, Crouzon case is included in the t-SNE embeddings, as with the complete models, this case again clusters among the Crouzon samples (Figure 3).

## **Over-4 Model Intrinsic Evaluation**

The reconstruction error for the face, head, and combined head-and-face models were 1.3 ± 1.2mm, 3.0 ± 2.3mm, and 2.3 ± 1.9mm, respectively. Notably, these values are lower than the reconstruction errors observed for the complete model even though a reduced number of samples has been used for model construction. We hypothesise that this is due to the reduced variation within the dataset given that the younger samples have been omitted. Specificity values of 1.8mm, 3.6mm, and 3.4mm for the face-only, head-only, and combined models, respectively. Again, these values are lower than for the complete models.

## **Over-4 Model Manifold Visualisation**

On applying t-SNE to the latent vectors for the samples in the Over-4 dataset, we again observe clusters forming for each of the included groups (Figure 4). This is clearest for the face-only model, however syndromic samples do still cluster separately from the healthy samples in both the head-only and combined head and face models. Though there are few Muenke samples in this cohort (n=2), we note that these still cluster separately from the normal population.

## **Over-4 Syndrome Classification**

As with the combined and Under-4 models, binary classification accuracies for the Over-4 model are greater than 99%. Again, high sensitivity and specificity values are recorded (Table 2).

Accuracies of 99.9%, 95.8%, and 96.2% were recorded for the multi-class classification experiments. From these results it is again observed that models that incorporate the face yield higher classification accuracies, further indicating the importance of the facial region for the classification of SC. Confusion matrices and multi-class classification matrices for the Over-4 models are shown in Figure 5.
